# Assessing genotype × environment interaction in linkage mapping using affected sib pairs

**DOI:** 10.1186/1753-6561-1-s1-s71

**Published:** 2007-12-18

**Authors:** Yi-Shin Chen, Yen-Feng Chiu, Hui-Yi Kao, Fang-Chi Hsu

**Affiliations:** 1 Department of Nursing, Yuanpei University, No. 306, Yuanpei Street, Hsinchu 30015, Taiwan, Republic of China; 2 Division of Biostatistics and Bioinformatics, National Health Research Institutes, 35 Keyan Road, Zhunan, Miaoli 350 Taiwan, Republic of China; 3 Department of Biostatistical Sciences, Wake Forest University School of Medicine, Medical Center Boulevard, Winston-Salem, North Carolina 27157, USA

## Abstract

Rheumatoid arthritis (RA) is a complex disease that involves both environmental and genetic factors. Elucidation of the basic etiologic factors involved in RA is essential for preventing and treating this disease. However, the etiology of RA, like that of other complex diseases, is largely unknown. In the present study, we conducted autosomal multipoint linkage scans using affected sib pairs by incorporating the smoking status into analysis. We divided the affected sib pairs into three subgroups based on smoking status (ever, current, or never). Interactions between the susceptibility genes and smoking could then be assessed through linkage mapping. Results suggested that the genetic effect of chromosome 6p21.2-3 in concordant current smoker pairs was about two-fold greater than that of the concordant non-current smoker pairs or discordant pairs. With incorporation of smoking status, additional regions with evidence of linkage were identified, including chromosomes 4q and 20q; while evidence of linkage remained in the regions of chromosomes 6p, 8p, and 9p. The interaction effects varied in different regions. Results from our analyses suggested that incorporating smoking status into linkage analyses could increase the statistical power of the multipoint linkage approach applied here and help elucidate the etiology of RA.

## Background

Numerous epidemiologic studies have shown that both genetic and environmental factors contribute to the development of rheumatoid arthritis (RA). Few studies have proceeded further to study how environmental and genetic factors might interact in individuals with RA [1]. To prevent and treat RA, it is necessary to understand the basic etiologic factors involved. Up to now, the most solid evidence for environmental influences exists for smoking; however, to the best of our knowledge, no previous studies have incorporated the effects of smoking into a genome-wide search for the susceptibility loci of RA. Because a gene × smoking interaction is likely to play an important role in the etiology of RA, incorporating smoking data to allow for interaction in linkage analysis would allow the interaction to be assessed and increase power for localizing a disease gene [2].

Recently, Liang et al. [3-5] proposed a robust multipoint linkage analysis approach using affected sib pairs by incorporating the information from environmental factors, which simultaneously tests for statistical interaction between the susceptibility locus and the environmental factors. It also provides estimates of the genetic effects stratified by the environmental factor and location of the disease locus τ, along with sampling uncertainty, to help investigators to narrow down chromosomal regions of interest. The value (1+C_*i*_)/2 (where the genetic effect for stratum *i *is denoted by "C_*i*_") characterizes the probability of an affected sib pair in stratum *i *sharing the same allele at τ from the parent. The genetic effects from the susceptibility locus for all stratified groups could be estimated, and the significance levels of these genetic linkage effects could be assessed at the estimated putative disease locus. Further, the significance of the interaction between a gene and smoking can be assessed through testing the null hypothesis, where all *C *values are considered equal. Hence, we adopted this multipoint linkage approach to assess the gene × smoking interaction for RA in the present study.

## Methods

### Materials

A total of 1096 affected sib pairs from 757 multiplex families in the North American Rheumatoid Arthritis Consortium (NARAC) study were included in the study. Only 615 or 627 sib pairs (depending on the chromosomal regions) had genotype information, and thus these were used for our analysis. The NARAC multiplex families contain 8017 individuals, most of whom are Caucasians (90.6%). We performed the analyses using the entire data set and the subset of Caucasians and found that the results from both data sets were virtually identical. We therefore reported only the results from the entire data set here. A total of 375 microsatellite markers were used in the analyses. There were 615 affected sib pairs available for chromosomes 1–11, 13–16, and 19–22, and 627 affected sib pairs available for chromosomes 12, 17, and 18. The smoking variables included "ever smoker" and "current smoker." Due to the missingness of smoking variables, the total number of affected sib pairs included in the analysis varied from 585 to 597, depending on which smoking variable was used, and which chromosomal region was studied.

Several studies have shown that the association between current heavy smokers and RA was striking, while the association between "ever smokers" and RA was modest (e.g., [6]). To understand the etiology of RA, it is therefore helpful to examine the interactions between these two smoking statuses and the trait locus of RA. The affected sib pairs were stratified according to their smoking status. The gene × smoking effect was examined separately for the two smoking variables. The three groups of affected sib pairs stratified by "ever smoked" status were (never smoked, never smoked) pairs, (never smoked, ever smoked) pairs, and (ever smoked, ever smoked) pairs; they were (non-current smoker, non-current smoker) pairs, (non-current smoker, current smoker) pairs, and (current smoker, current smoker) pairs when stratified by the "current smoking" status. For chromosomes 12, 17, and 18, the numbers of (never smoked, never smoked), (never smoked, ever smoked), and (ever smoked, ever smoked) affected sib pairs were 163, 206, and 225, respectively; there were 160, 203, and 222, respectively, for the rest of chromosomes. In addition, the numbers of affected sib pairs for (non-current smoker, non-current smoker), (non-current smoker, current smoker), and (current smoker, current smoker) were 425, 138, and 34, respectively, for chromosomes 12, 17, and 18, and 417, 137, and 34, respectively, for the rest of chromosomes. Among the 425 (417) concordant "non-current smoker" pairs, 160 (163) of them were concordant "never smoked" pairs, while 34 (34) out of 225 (222) "ever smoked" pairs were "current smoker" pairs. There were 636 (about 39.9%) former smokers who were "ever smokers", yet were not "current smokers." Five affected sibs (about 0.31%) mistakenly reported that they never smoked, yet were current smokers. The difference in numbers of the affected sib pairs defined by never/ever smoked and non-current/current smokers was made by these 641 (636+5) affected sibs.

### Statistical methods

The parameters *C*_0_, *C*_1_, and *C*_2 _were the genetic effects for the three groups stratified by one of the smoking statuses, respectively. The GeneHunter program was used to calculate identity-by-decent (IBD) sharing of affected sib pairs. The GeneFinder program was applied to obtain the estimates of τ and *C*_*i*_, *i *= 0, 1, 2, and their 95% confidence intervals, as well as to calculate the *p*-values of the genetic effects to test whether C values were all equal (that is, if the gene × environment interaction was present). In addition, we compared these results with the results from analyses excluding environmental factors.

## Results

**Figure 1:**
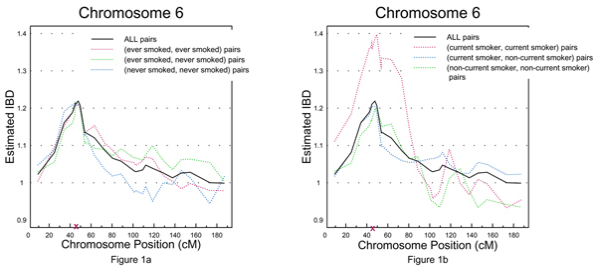
a, Estimated IBD stratified by "ever smoked" vs. overall estimated IBD for chromosome 6; b, estimated IBD stratified by "current smoker" vs. overall estimated IBD for chromosome 6.

For comparison, we demonstrated the results from the autosomal-wide scan in which smoking status was not incorporated in Table 1. As illustrated in Table 2 and Figure 1a, after stratifying on the status of "ever smoked," the susceptibility disease locus on chromosome 6 remained in the same region as that identified without incorporating "ever smoked" status, around 45.6 cM on chromosome 6. The genetic effects from the three "ever smoked" groups remained statistically significant and were similar at this locus, with $\hat{C}$_0 _= 0.21 (*p *= 0.00012), $\hat{C}$_1 _= 0.20 (*p *= 0.000031) and $\hat{C}$_2 _= 0.22 (*p *= 2.32 × 10^-6^). Therefore, the interaction between the susceptibility locus and "ever smoked" status was not statistically significant (*p *= 0.95). Nevertheless, the interaction of the gene by "ever smoked" status was observed at 25.84 cM on chromosome 8 (*p *= 0.0055), at 23.9 cM on chromosome 13 (*p *= 0.026), at 55.12 cM on chromosome 15 (*p *= 0.029), and at 44.85 cM on chromosome 17 (*p *= 0.017). The (never smoked, never smoked) and (never smoked, ever smoked) pairs showed significant genetic effects ($\hat{C}$_0 _= 0.22, *p *= 0.00047; $\hat{C}$_1 _= 0.10, *p *= 0.027) at the susceptibility locus identified on chromosome 8, but not on chromosomes 13, 15, or 17, indicating that the genetic effect of "ever smoked" status varied from region to region, and the interaction could still exist when the genetic effect for each stratum in the region was not statistically significant.

**Table 1:** Autosomal-wide linkage mapping without incorporating a smoking variable

Chr	$\hat{\tau }$ (SE)	95% C.I. for τ	$\hat{C}$ (SE)	*p*-Value for testing *H*_0_*: C = 0*
1	142.81 (13.6)	[116.16,169.46]	0.04 (0.031)	0.095
2	207.79 (12.39)	[183.52,232.07]	0.032 (0.03)	0.14
3	24.24 (14.03)	[-3.26,51.74]	0.023 (0.029)	0.21
4	178.47 (53.35)	[73.91,283.02]	-0.006 (0.03)	1
5	91.6 (8.49)	[74.96,108.24]	0.063 (0.036)	0.04
6	45.87 (1.26)	[43.39,48.34]	0.219 (0.028)	3.80 × 10^-15^
7	90.37 (36.11)	[19.59,161.14]	0.014 (0.036)	0.35
8	7.09 (4.78)	[-2.28,16.46]	0.08 (0.029)	0.0027
9	40.11 (6.82)	[26.74,53.48]	0.067 (0.033)	0.02
10	84.21 (8.01)	[68.51,99.91]	0.049 (0.029)	0.047
11	67.67 (25.9)	[16.9,118.43]	0.034 (0.045)	0.22
12	52.16 (13.49)	[25.72,78.59]	0.036 (0.032)	0.13
13	90.07 (17.77)	[55.24,124.9]	-0.022 (0.028)	1
14	49.75 (15.29)	[19.78,79.71]	0.032 (0.037)	0.19
15	60.55 (6.24)	[48.31,72.78]	-0.042 (0.027)	1
16	49.83 (6.90)	[36.3,63.36]	0.064 (0.035)	0.034
17	23.39 (36.58)	[-48.31,95.09]	-0.01 (0.027)	1
18	70.22 (8.80)	[52.97,87.47]	0.056 (0.033)	0.043
19	82.32 (4.43)	[73.63,91.01]	-0.098 (0.027)	1
20	18.75 (10.66)	[-2.15,39.64]	-0.059 (0.038)	1
21	Not Convergent			
22	34.74 (4.94)	[25.06,44.42]	-0.043 (0.027)	1

**Table 2:** Incorporating "ever smoked" status into linkage mapping using affected sib pairs

		Estimate (*p*-Value)			
		
Chr	$\hat{\tau }$ (SE)	$\hat{C}$_0_	$\hat{C}$_1_	$\hat{C}$_2_	*T*esting *H*_0_: *C*_0 _= *C*_1 _= *C*_2 _= *C*
1	120.04 (7.60)	-0.046 (0.45)	0.058 (0.29)	0.068 (0.14)	0.28
2	25.54 (6.94)	-0.086 (0.12)	-0.047 (0.31)	-0.041 (0.36)	0.79
3	216.54 (4.60)	0.021 (0.71)	-0.090 (0.066)	-0.092 (0.046)	0.22
4	99.56 (7.25)	-0.039 (0.55)	0.100 (0.085)	-0.037 (0.48)	0.15
5	44.83 (7.60)	-0.030 (0.63)	0.082 (0.11)	0.030 (0.57)	0.37
6	45.6 (1.34)	0.212 (1.2 × 10^-4^)	0.200 (3.1 × 10^-5^)	0.222 (2.3 × 10^-6^)	0.95
7	32.96 (7.10)	0.033 (0.57)	-0.036 (0.47)	-0.059 (0.23)	0.46
8	25.84 (2.86)	0.217 (4.7 × 10^-4^)	0.104 (0.027)	-0.035 (0.49)	0.0055
9	41.67 (6.72)	0.070 (0.25)	0.040 (0.50)	0.086 (0.13)	0.85
10	25.02 (6.19)	0.084 (0.16)	-0.032 (0.50)	-0.047 (0.32)	0.18
11	83.73 (6.78)	-0.062 (0.29)	0.019 (0.71)	0.077 (0.12)	0.19
12	115.95 (6.01)	0.066 (0.37)	0.025 (0.70)	-0.159 (0.0022)	0.12
13	23.9 (5.53)	0.102 (0.089)	-0.097 (0.057)	-0.065 (0.17)	0.026
14	43.04 (8.95)	-0.015 (0.79)	-0.008 (0.87)	0.077 (0.24)	0.5
15	55.12 (3.80)	-0.091 (0.069)	0.066 (0.20)	-0.099 (0.028)	0.029
16	62.59 (5.18)	0.027 (0.66)	0.085 (0.13)	0.074 (0.15)	0.76
17	44.85 (5.65)	-0.151 (0.012)	0.049 (0.40)	0.052 (0.30)	0.017
18	118.42 (4.06)	0.035 (0.52)	-0.101 (0.032)	0.005 (0.92)	0.12
19	83.74 (3.79)	-0.025 (0.65)	-0.148 (0.0037)	-0.133 (0.0031)	0.20
20	50.23 (8.23)	0.009 (0.88)	0.004 (0.94)	-0.070 (0.14)	0.47
21	30.09 (6.34)	0.028 (0.61)	0.032 (0.44)	-0.042 (0.29)	0.37
22	28.76 (4.62)	-0.018 (0.76)	-0.068 (0.16)	-0.036 (0.44)	0.79

When stratified by "current smoking" status (Table 3), the susceptibility disease locus on chromosome 6 remained at the same location, and the interaction between this locus and current smoking status was statistically significant (*p *= 0.023). The genetic effect from the (current smoker, current smoker) group was estimated to be 0.43 (*p *= 0.0018), about two-fold higher than those from the (non-current smoker, non-current smoker) ($\hat{C}$_0 _= 0.20, *p *= 1.30 × 10^-8^) and (non-current smoker, current smoker) ($\hat{C}$_1 _= 0.20, *p *= 0.00068) groups as illustrated in Figure 1b.

**Table 3:** Incorporating "current smoker" status into linkage mapping using affected sib pairs

		Estimate (*p*-Value)			
		
Chr	$\hat{\tau }$ (SE)	$\hat{C}$_0_	$\hat{C}$_1_	$\hat{C}$_2_	*T*esting *H*_0_:*C*_0 _= *C*_1 _= *C*_2 _= *C*
1	50.96 (5.79)	-0.042 (0.20)	0.087 (0.17)	-0.022 (0.87)	0.15
2	94.63 (6.38)	-0.056 (0.12)	-0.076 (0.19)	0.019 (0.86)	0.61
3	216.59 (5.58)	-0.054 (0.12)	-0.083 (0.15)	-0.082 (0.54)	0.85
4	160.24 (6.73)	-0.040 (0.27)	0.121 (0.044)	-0.105 (0.34)	0.045
5	188.91 (8.03)	-0.057 (0.11)	-0.025 (0.70)	0.090 (0.53)	0.19
6	45.76 (1.29)	0.199 (1.3 × 10^-8^)	0.197 (6.8 × 10^-4^)	0.425 (0.0018)	0.023
7	32.68 (5.60)	0.000 (1.00)	-0.126 (0.030)	0.038 (0.76)	0.15
8	26.31 (3.43)	0.119 (9.5 × 10^-4^)	0.002 (0.97)	-0.087 (0.51)	0.0086
9	38.79 (4.62)	0.110 (0.0060)	0.013 (0.86)	-0.213 (0.064)	0.00052
10	26.94 (6.05)	-0.012 (0.72)	-0.034 (0.58)	0.200 (0.10)	0.033
11	9.04 (4.87)	-0.043 (0.18)	0.084 (0.15)	-0.001 (0.99)	0.10
12	51.51 (8.85)	0.037 (0.37)	0.087 (0.18)	-0.081 (0.51)	0.34
13	37.99 (5.64)	-0.016 (0.63)	-0.103 (0.071)	0.166 (0.094)	0.023
14	83.04 (9.72)	-0.014 (0.74)	0.032 (0.72)	0.207 (0.087)	0.052
15	14.95 (8.02)	-0.026 (0.42)	-0.082 (0.14)	-0.028 (0.82)	0.66
16	58.61 (5.20)	0.041 (0.28)	0.090 (0.20)	0.200 (0.19)	0.15
17	111.99 (10.07)	-0.013 (0.76)	0.042 (0.58)	-0.164 (0.26)	0.28
18	74.46 (6.68)	0.070 (0.073)	0.031 (0.63)	-0.178 (0.19)	0.018
19	81.12 (3.43)	-0.108 (0.0015)	-0.036 (0.56)	-0.319 (2.1 × 10^-4^)	0.0096
20	88.46 (4.00)	-0.002 (0.96)	-0.046 (0.50)	0.214 (0.052)	0.040
21	38.43 (4.59)	0.007 (0.83)	0.025 (0.59)	-0.165 (0.14)	0.052
22	37.41 (4.14)	-0.039 (0.25)	-0.077 (0.16)	-0.150 (0.18)	0.29

Other regions showed a significant interaction between the susceptibility locus and current smoking status, including the locations of 160.2, 26.3, 38.8, 26.9, 38.0, 83.0, 74.5, 81.1, 88.5, and 38.4 cM on chromosomes 4, 8, 9, 10, 13, 14, 18, 19, 20, and 21, respectively. The gene × smoking interactions were most striking on chromosomes 8 (*p *= 0.0086) and 9 (*p *= 0.00052). Among them, the (non-current smoker, non-current smoker) group showed statistically significant genetic effects on chromosomes 8p and 9p with $\hat{C}$_0 _= 0.12 (*p *= 0.00095) and 0.11 (*p *= 0.0060), respectively. The (non-current smoker, current smoker) group showed a statistically significant genetic effect on chromosome 4q ($\hat{C}$_1 _= 0.12, *p *= 0.044), and the (current smoker, current smoker) group showed a statistically significant genetic effect on chromosomes 20q ($\hat{C}$_2 _= 0.21, *p *= 0.052).

## Conclusion

The etiology of RA is multifactorial, with genetic factors contributing between 30 and 50% of the total risk [6]. We conducted genome-wide multipoint linkage scans by stratifying on the status of "ever smoked" or "current smoker" to assess the interaction between smoking and the susceptibility locus for RA. The "current smoker" status significantly interacted with the locus identified in the region of 6p21.2-3, while "ever smoked" did not. The genetic effect in this region for "current smoker" pairs was about two-fold of that for other types of pairs, suggesting that smoking might trigger genes with immunologic significance in this region more strongly in current smokers than it does for "non-current" smokers.

It is estimated that about one-third of the total proportion of the genetic risk arises from the major histocompatibility complex that lies in the 6p21.2-3 region, which is known to contain more than 220 genes [7]. Our results suggested that the genes in this region interact with current smoking status. In addition, by incorporating smoking status into our analyses, we also identified other regions with statistically significant genetic effects and interaction effects between current smoking status and the susceptibility locus, including regions on chromosomes 4q, 8p, 9p, and 20q. Among them, the concordant non-current smoker pairs showed stronger genetic effects on chromosomes 8p and 9p, while the concordant current smoker pairs showed stronger genetic effects on chromosome 20q. These findings help investigators to dissect the etiology and underlying genetic mechanism of RA, which is critical for designing new tools for suppressing RA pathogenesis before the onset of disease.

## Competing interests

The author(s) declare that they have no competing interests.
